# Acute Circadian Disruption Due to Constant Light Promotes Caspase 1 Activation in the Mouse Hippocampus

**DOI:** 10.3390/cells12141836

**Published:** 2023-07-12

**Authors:** Pikria Ketelauri, Katerina Scharov, Charlotte von Gall, Sonja Johann

**Affiliations:** 1 Institute of Anatomy II, Medical Faculty, Heinrich-Heine-University (HHU), 40225 Düsseldorf, Germany; 2 Institute of Neuroanatomy, University Medical Center Hamburg-Eppendorf (UKE), Martinistraße 52, 20251 Hamburg, Germany

**Keywords:** circadian disruption, constant light, inflammasomes, caspase 1, interleukins, hippocampus

## Abstract

In mammals, the circadian system controls various physiological processes to maintain metabolism, behavior, and immune function during a daily 24 h cycle. Although driven by a cell-autonomous core clock in the hypothalamus, rhythmic activities are entrained to external cues, such as environmental lighting conditions. Exposure to artificial light at night (ALAN) can cause circadian disruption and thus is linked to an increased occurrence of civilization diseases in modern society. Moreover, alterations of circadian rhythms and dysregulation of immune responses, including inflammasome activation, are common attributes of neurodegenerative diseases, including Alzheimer’, Parkinson’s, and Huntington’s disease. Although there is evidence that the inflammasome in the hippocampus is activated by stress, the direct effect of circadian disruption on inflammasome activation remains poorly understood. In the present study, we aimed to analyze whether exposure to constant light (LL) affects inflammasome activation in the mouse hippocampus. In addition to decreased circadian power and reduced locomotor activity, we found cleaved caspase 1 significantly elevated in the hippocampus of mice exposed to LL. However, we did not find hallmarks of inflammasome priming or cleavage of pro-interleukins. These findings suggest that acute circadian disruption leads to an assembled “ready to start” inflammasome, which may turn the brain more vulnerable to additional aversive stimuli.

## 1. Introduction

Circadian rhythms are endogenous, autonomous, and self-sustained oscillations maintaining the behavioral and physiological rhythms of virtually all organisms [1]. In mammals, circadian rhythms control nearly all kinds of physiological processes, including sleep, cognition, locomotor activity, energy metabolism, and immune response [2,3,4,5]. Increasing evidence suggests that immune responses, such as immune cell trafficking, phagocytic capacity, and expression and secretion of inflammatory molecules, are also controlled by the circadian clock [6,7]. Endogenous circadian rhythms can be entrainment to external or environmental cues (*Zeitgeber*), including light–dark cycle, feeding, and temperature [3,8] synchronizing internal time to the earth’s 24 h rotation. In the absence of a *Zeitgeber*, circadian rhythms will run freely with a rhythm that can considerably deviate from a 24 h day [9]. The mammalian circadian system consists of a central pacemaker, the suprachiasmatic nucleus (SCN), localized in the hypothalamus. The SCN entrains subordinated clocks in the brain and in the periphery through the rhythmic activity of the autonomous nervous system and rhythmic hormones such as melatonin and glucocorticoids [10]. The SCN oscillator itself is entrained by the environmental light–dark cycle through projections from the retina [10,11,12]. At the cellular level, rhythms in gene expression and, thus, cell functions are regulated by a transcription–translation feedback loop, highly conserved across animal species [13,14].

Abnormal light exposure, such as light at night and constant light (LL), can disrupt circadian rhythms and cause desynchronization in central and peripheral clocks. Thus, LL disrupts circadian rhythms of spontaneous activity, plasma melatonin, and corticosterone [15,16], induces depressive-like behavior [17], exacerbates inflammatory responses to pathogenic stimuli [18,19], and impairs survival during recovery from sepsis [20]. Alterations in circadian rhythms, such as disturbances of the sleep/wake rhythm, are common in aging subjects and even more severe in patients suffering from metabolic, cardiovascular, and neurodegenerative diseases [21]. Irregularities in circadian rhythmicity are early signs in neurodegenerative diseases, including Parkinson’s disease (PD) and Alzheimer’s disease (AD), and often occur prior to clinical diagnosis. Moreover, there seems to be a bidirectional relationship, as circadian disruption exacerbates the progression of neurodegenerative diseases [22,23,24].

Neuroinflammation is often associated with and significantly contributes to neurodegenerative diseases [25,26,27,28]. In recent years, the involvement of inflammasome activation in aging and the pathogenesis of numerous neurological diseases, including AD, PD, stroke, and amyotrophic laterals sclerosis (ALS), has been studied with particular interest [29,30,31,32,33]. Inflammasomes are multiprotein complexes classified by the type of cytoplasmatic pattern recognition receptor (PRR). The NOD-like receptor family, including NLRP1, NLRP3, and NLRC4, and absent in melanoma 2 (AIM2) containing a HIN200 and pyrin domain are among the most studied inflammasomes [34,35]. Assembly and activation of inflammasomes occur after the binding of pathogen-associated molecular patterns (PAMPs) and/or damage-associated molecular patterns (DAMPs) to the specific cytosolic PRRs. In the next step, pro-caspase 1 is recruited by the adaptor molecule apoptosis-associated speck-like protein containing a CARD (ASC) which promotes autoproteolytic cleavage of pro-caspase 1 and subsequent processing and secretion of IL1β and pro-IL18 [36,37].

It has long been known that the response of the innate immune system to pathogens shows a circadian rhythm [38,39]. Inflammasome components, such as NLRP3, IL1β, and IL18, oscillate in a daily manner in the brain [40,41] and immune cells [42]. Moreover, cytokines, including TNF and IL1β, feed back into the central and peripheral clocks and sleep regulatory centers [43] and were shown to affect locomotor activity by interacting with clock gene expression [44]. Recently, the clock gene protein Rev-erbα has been demonstrated to attenuate NLRP3-mediated neuroinflammation in bone marrow-derived macrophages [45] and microglia [46]. Taken together, these findings demonstrate that circadian rhythms and molecular clockwork are implicated in the regulation of inflammasome activation and vice versa. However, the direct effect of circadian disruption on the brain inflammasome remains unknown.

In the present study, we explored the impact of acute circadian disruption induced by constant light on inflammasome activation in the hippocampus. The hippocampus was chosen because it: (i) controls rhythmic cognitive functions; (ii) is affected by aging and neurological and mental diseases (e.g., AD, epilepsy, depression, schizophrenia); (iii) it responds to chronic stress with inflammasome activation [47]; (iv) is highly sensitive to adverse effects of neuroinflammation [48]; and (v) is easily accessible for preparation and has a well-known anatomy.

## 2. Materials and Methods

### 2.1. Animals

Twenty male C57Bl/6 mice (10 weeks of age) were obtained from Janvier Labs (Le Genest-Saint-Isle, France). Upon arrival, mice were randomly assigned into two groups (*n* = 10), weighted, and housed individually in cages with free access to food and water, equipped with infrared movement detectors linked to an automated recording system (Mouse-E-Motion, Hamburg, Germany). Animals were maintained for 10 days under a standard light/dark photoperiod of 12 h light (400 lux): 12 h darkness (<2 lux) (LD) in a soundproof cabinet with automatic control of the photoperiod (lights on at 6:00 AM and off at 6:00 PM) (Scanbur, Denmark, Karlslunde) to allow them to habituate to light settings and to recover from shipping. Subsequently, the control group was maintained under LD conditions while the experimental group was exposed to constant light (12:12 light/light (LL)) for 14 days. Samples were collected between 3 and 8 h after lights-on in LD and between 3 and 8 h after former lights-on in LL, thus within the same circadian phase. Before cardiac puncture and perfusion, mice were deeply anesthetized using ketamine:xylazine (100 mg:10 mg/kg body weight), and death was confirmed by a cessation of respiration and the absence of reflexes.

Animals were transcardially perfused with 0.9% NaCl to provide optimal quality for native tissue analysis, followed by perfusion with 4% paraformaldehyde for immunofluorescence studies. Tissue collection was executed during the light phase and included processing for rtPCR/Western blot analysis (LD *n* = 5, LL *n* = 5) and immunofluorescence (LD *n* = 5, LL *n* = 5). All animal experiments were performed under the terms of the German animal protection law and according to the regulations of the local animal research council and legislation of the State of North Rhine-Westphalia (case number: 84-02.04.2013.A358).

### 2.2. Analysis of Locomotor Activity Rhythms

Spontaneous locomotor activity was recorded in 10 min intervals using an infrared Universal Data Logger (Mouse-E-Motion, Hamburg, Germany) as described previously [49]. Actograms, chi-square periodogram analysis, fast Fourier transform (FFT) analysis, and activity profiles were calculated using Clocklab software Version 2.61, (Actimetrics, Wilmette, IL, USA) as previously described [49]. Total activity, circadian strength, and period length were estimated based on the observation period of 14 consecutive days in LD and/or in LL.

### 2.3. Preparation of Peripheral Blood Films and White Blood Cell (WBC) Counting

Blood collection was performed immediately before perfusion with 0.9% NaCl via cardiac puncture. A disposable 1 mL EDTA-coated syringe with a 25-gauge needle was inserted into the right ventricle. Between 0.5 and 0.75 mL of blood was collected from each animal. Blood films were prepared by dispensing a drop of blood onto one end of a clean microscope slide. A second slide was placed at an angle of 45° in front and rapidly pushed forward to create a monolayer smear for WBC counting. After air-drying, blood films were stained with May–Grunwald–Giemsa and mounted with Entellan^®^ (Merck, Germany). Per sample, 200 WBCs were counted under a light microscope (Leitz, Germany). The report is provided according to Schilling classification, and data are expressed as leukocyte type proportion (%).

### 2.4. RNA Isolation, Reverse Transcription (RT), and Real-Time PCR (rtPCR)

Gene expression study was performed using rtPCR technology (StepOnePlus Real-Time PCR System, Thermo Fisher, Waltham, MA, USA), KAPA SYBR^®^ FAST qPCR Master Mix (2X) Kit (Kapa Biosystems, Cape Town, South Africa). Briefly, tissue was rapidly homogenized using the Precellys Evolution tissue homogenizer (Bertin Technologies, Montigny-le-Bretonneux, France), and extraction of total RNA was performed with peqGOLD TriFast^TM^ (VWR Life Science; Darmstadt, Germany). RNA samples (1 µg) were treated with DNase1 (Roche, Mannheim, Germany) to eliminate genomic DNA. Reverse transcription was conducted using the M-MLV RT-kit (Thermo Fisher Scientific, Waltham, MA, USA) and hexanucleotide primers. The qBase + software package Version 3.4 was applied to assign suitable reference genes and to estimate relative gene expression. *GAPDH* and *HSP90* served as internal controls. Primer sequences (Table 1) were designed using Primer3web version 4.1.0 (https://primer3.ut.ee/; accessed on 1 July 2022) [50] or obtained from PrimerBank (https://pga.mgh.harvard.edu/primerbank/; accessed on 1 July 2022) [51]. Finally, data of the experimental group (LL) were expressed as a fold of the control group (LD). Melting curves were analyzed to control for primer specificity.

### 2.5. Immunoblot Analysis

Frozen tissue was homogenized in ice-cold RIPA buffer as described previously [52] using PRECELLYS^®^ Evolution tissue homogenizer (Bertin Instruments, Montigny-le-Bretonneux, France). The BCA Protein Assay (Thermo Fisher Scientific, Waltham, MA, USA) was applied according to the manufacturer’s protocol to determine protein concentration. Protein samples (20 μg per lane) were separated using SDS-PAGE and transferred to a polyvinylidene difluoride or nitrocellulose membrane (Roche Diagnostics, Mannheim, Germany). After blocking, membranes were incubated with specific primary antibodies overnight at 4 °C (see Table 2 for antibody details). The following day, membranes were labeled with the appropriate horseradish peroxidase-conjugated secondary antibody for 1–2 h at room temperature. After washing, visualization was performed using Immobilon ECL (Millipore, Burlington, MA, USA) and Chemi Only Gel Documentation System (VWR). GAPDH and vinculin served as internal control. A densitometric evaluation was conducted using ImageJ software Version 1.53e (free Java software provided by the National Institutes of Health, Bethesda, MD, USA). Finally, data from the experimental group (LL) were expressed as a fold of the control group (LD).

### 2.6. Immunofluorescence

Immunofluorescence (IF) was performed on paraffin-embedded 5 µm thick cross-sections as previously described [52]. Brain sections were rehydrated and heat-unmasked for 20 min using citrate buffer (pH 6). After blocking with 5% normal serum, sections were incubated with IBA1 (1:2000, Fujifilm Wako, Neuss, Deutschland, 019-19741) and GFAP (1:500, Dako, Z0334) overnight at 4 °C (Table 2). Secondary antibodies, either conjugated with the fluorescent dye Alexa 488 or 568 (1:500; Invitrogen, Germany), were applied for 1 h at RT. Cell nuclei were counterstained with Hoechst 33342 (1:10,000; Invitrogen, Germany). Autofluorescence was minimized by incubation with 0.3% Sudan Black solved in 70% ethanol for 5 min. Sections were viewed using a Keyence Fluorescence Microscope (BZ-900 E BIOREVO, Osaka, Japan), and images were processed by BZ-II analyzer software Version 2.2 (Keyence, Osaka, Japan). Two brain sections per animal were analyzed (approximately −1.96 mm and −2.26 mm from bregma). Fluorescence pictures of the right and left hippocampi were taken for analysis. ImageJ software was applied to determine the mean fluorescence intensity (MFI) of the hippocampal formation, including the hippocampus proper and dentate gyrus. Finally, data from LL were expressed as a fold of LD.

### 2.7. Statistical Analysis

Data were evaluated using GraphPad Prism 5.0 and IBM SPSS Statistics 22 software. Parametric statistics were applied with datasets with at least *n* = 8 per group that met Shapiro–Wilk criteria for normal distribution and homogeneity of variances using Levene’s test (most behavioral data, weight, white blood cell count; *n* = 8–10 per group). Small datasets (*n* = 5) or data with non-normal distributions were analyzed by the non-parametric Mann–Whitney U test for comparison of mean differences between two groups. Differences were considered significant when *p* ≤ 0.05. For better visualization, data from rtPCR, Western blotting, and immunofluorescence were normalized by dividing the complete dataset by the mean of the LD control group, resulting in a mean of 1 for LD and a relative to 1 value for the LL group. Box and whisker plots indicating all data points were prepared to graphically depict data. The boxes represent 25–75% of the range; the whiskers indicate the minimum and maximum values, and the median is shown by a vertical line.

## 3. Results

### 3.1. Constant Light Significantly Affects Rhythmic Activity but Not Body Weight and White Blood Cell Composition

**Figure 1:**
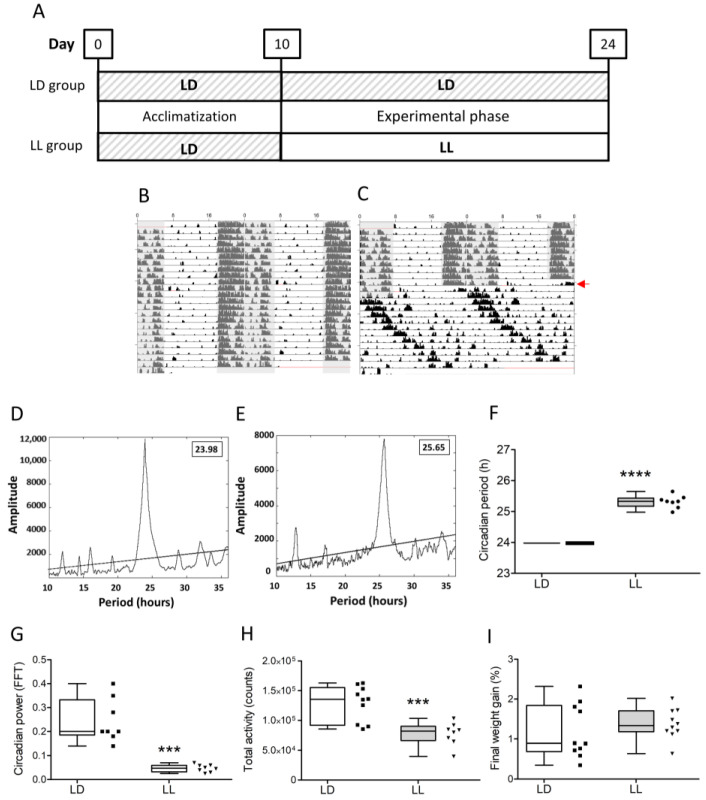
Constant light significantly disrupts circadian rhythms in behavior but does not affect weight gain proportion of different leukocyte subsets. (**A**) Experimental protocol. Representative double-plotted actograms and periodograms of an LD (**B**,**D**) and LL mouse (**C**,**E**). Gray shading indicates periods of darkness, and the red arrow (**C**) indicates the start of the experimental phase with constant light for the LL group. Circadian period (**F**) was increased, whereas circadian power (**G**) and total activity (**H**) were significantly reduced in LL mice. Final weight gain (**I**) was not different between groups. Data were analyzed using 2-tailed unpaired Student’s *t*-test, *** *p* < 0.001, and Mann–Whitney U tests, **** *p* < 0.0001.

After 10 days of acclimatization, the control group was maintained under LD conditions while the experimental group was exposed to LL for 14 days (Figure 1A). The spontaneous locomotor activity of mice was continuously recorded during acclimatization and the experimental phase of LD (Figure 1B) or LL (Figure 1C). The period of behavioral rhythm was significantly shorter in LD, 23.98 ± 0.0 (Figure 1D,F) than in LL, 25.32 ± 0.2 (Figure 1E,F) (Mann–Whitney U = 80.00, n1 = 10, n2 = 8, **** *p* = <0.0001, two-tailed). All mice under LL were classified as “rhythmic” by F-periodogram. However, in mice under LL strength of rhythmicity was significantly lower (t(14) = 6.042, *** *p* = 0.0001, two-tailed) compared to LD (Figure 1G). Furthermore, in mice under LL, total activity was significantly lower (t(16) = 4.216, *** *p* = 0.0007 two-tailed), approximately 60%, than of mice in LD (Figure 1H). Final weight gain was not different (t(9) = 0.6228, *p* = 0.5451, two-tailed) (Figure 1I).

**Figure 2:**
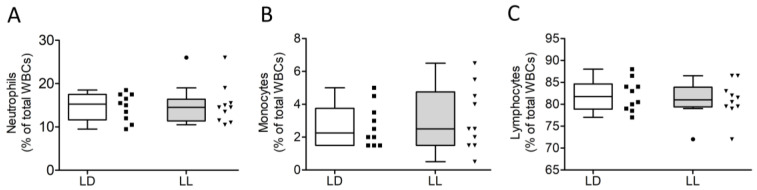
Constant light did not affect the proportion of different leukocyte subsets. Percentages of different WBC types in LD and LL. (**A**) Neutrophils (%), (**B**) monocytes (%), and (**C**) lymphocytes (%). Data were analyzed using 2-tailed unpaired Student’s *t*-test and Mann–Whitney U test.

Leukocyte composition was analyzed as a marker for stress and general inflammatory response. We did not find significant differences in percentage of neutrophils (Figure 2A; Mann–Whitney U = 47, n1 = n2 = 10, *p* = 0.8498, two-tailed), monocytes (Figure 2B; t(18) = 0.5449, *p* = 0.5925, two-tailed) or lymphocytes (Figure 2C; (t(18) = 0.6026, *p* = 0.5543, two-tailed) between LD and LL. Due to the very low percentage (<1%), eosinophils and basophils were not statistically analyzed.

### 3.2. Exposure to LL Mildly Affects Glial Activation

**Figure 3:**
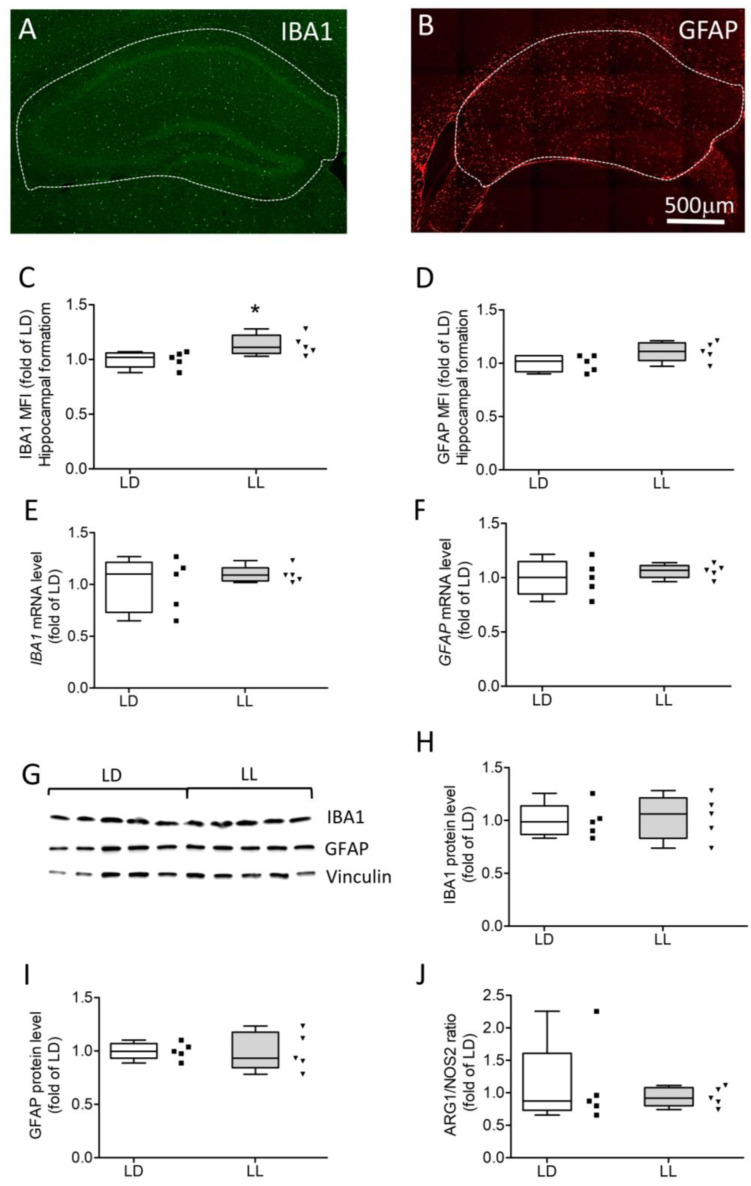
Mild glial activation after two weeks of constant light. Representative image of immunostaining for the microglial marker IBA1 (**A**) and the astrocyte marker GFAP (**B**) in the hippocampus of LL mice. Mean fluorescence intensity of IBA1 (**C**) but not GFAP (**D**) was significantly increased in the LL group. Transcription levels (**E**,**F**) and protein concentration (**G**–**I**) of both markers are similar between groups. No difference in ARG1/NOS2 mRNA ratio (**J**) was detected. Data were analyzed using Mann–Whitney U tests. * *p* < 0.05.

In response to danger signals, glial cells adopt an activated phenotype resulting in morphological changes and the release of pro-inflammatory mediators. Hence, we investigated microglia/macrophages and astrocytes using the respective markers IBA1 (Figure 3A) and GFAP (Figure 3B). The MFI of both glial markers was slightly increased in LL, but only IBA1 was found to be significantly different (Figure 3E Mann–Whitney U = 2.00, n1 = n2 = 5, *p* = 0.0317 two-tailed). However, mRNA levels (Figure 3E,F) and protein concentrations (Figure 3G–I) were similar in both groups. Additionally, changes in microglia’s M1/M2 state were studied by analyzing the ratio of the M1 marker, nitric oxide synthases 2 (NOS2), and M2 marker, arginase 1 (ARG1). Since ARG1 and NOS2 compete for the same substrate, L-arginine, changes in the transcriptional ratio of these markers can indicate M1 or M2 polarization. The ARG1/NOS2 ratio was not different between the two groups (Figure 3J). Taken together, these data suggest a mild microglia reactivity without evidence of a change in the activation state.

### 3.3. Constant Light Induces Cleavage of Caspase 1

In response to PAMPs/DAMPs, inflammasome complexes assemble in the cytoplasm. For some inflammasomes, such as NLRP3, a two-step process comprising priming and activation is required [53]. To evaluate inflammasome priming and activation, we performed rtPCR and Western blot analysis.

**Figure 4:**
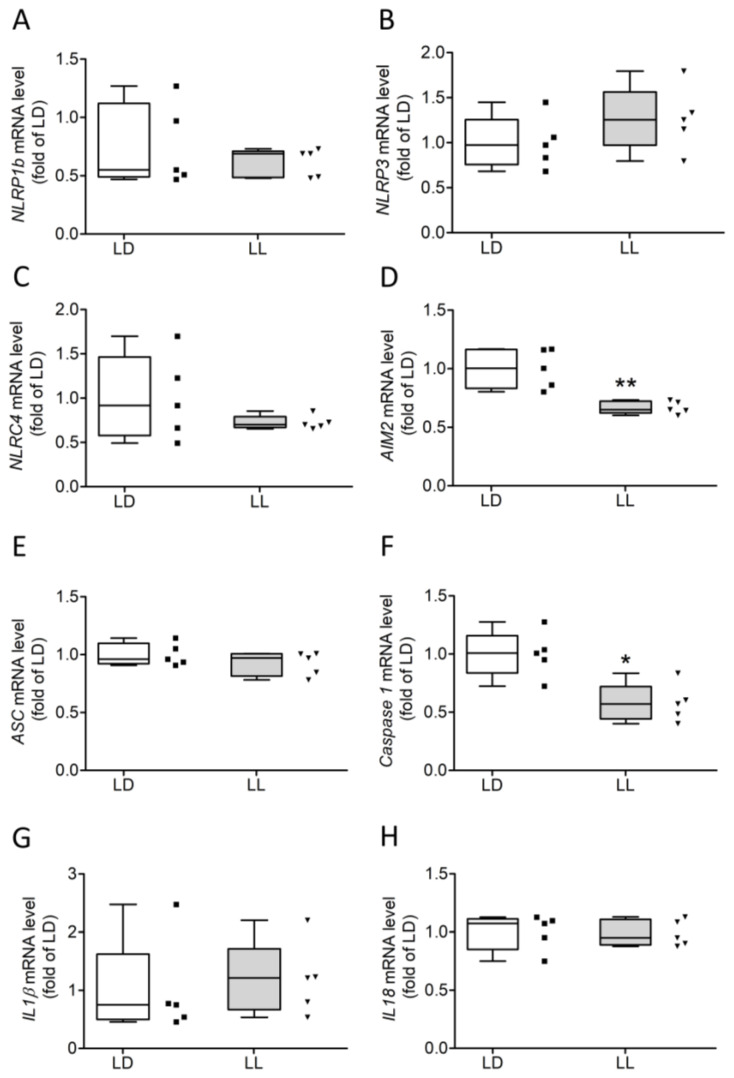
Transcription levels of inflammasome components were only slightly affected in LL mice. Transcription levels of inflammasome components (**A**–**F**) and interleukins (**G**,**H**) revealed a significant reduction in *AIM2* (**D**) and *Caspase 1* (**F**) in LL mice. Transcription levels of *NLRP1b* (**A**), *NLRP3* (**B**), *NLRC4* (**C**), *ASC* (**E**), *IL1β* (**G**), and *IL18* (**H**) were unaffected. Data were analyzed using Mann–Whitney U tests. * *p* < 0.05, ** *p* < 0.001.

Relative mRNA levels of *AIM2* (Figure 4D; Mann–Whitney U = 0.00, n1 = n2 = 5, *p* = 0.0079 two-tailed) and *Caspase 1* (Figure 4F; Mann–Whitney U = 1.00, n1 = n2 = 5, *p* = 0.0159 two-tailed) were significantly lower in the LL group. No differences in mRNA levels were detected for the PRRs *NLRP1b* (Figure 4A), *NLRP3* (Figure 4B), and *NLRC4* (Figure 4D), for *ASC* (Figure 4E), or for the interleukins *IL1β* (Figure 4G) and *IL18* (Figure 4H).

**Figure 5:**
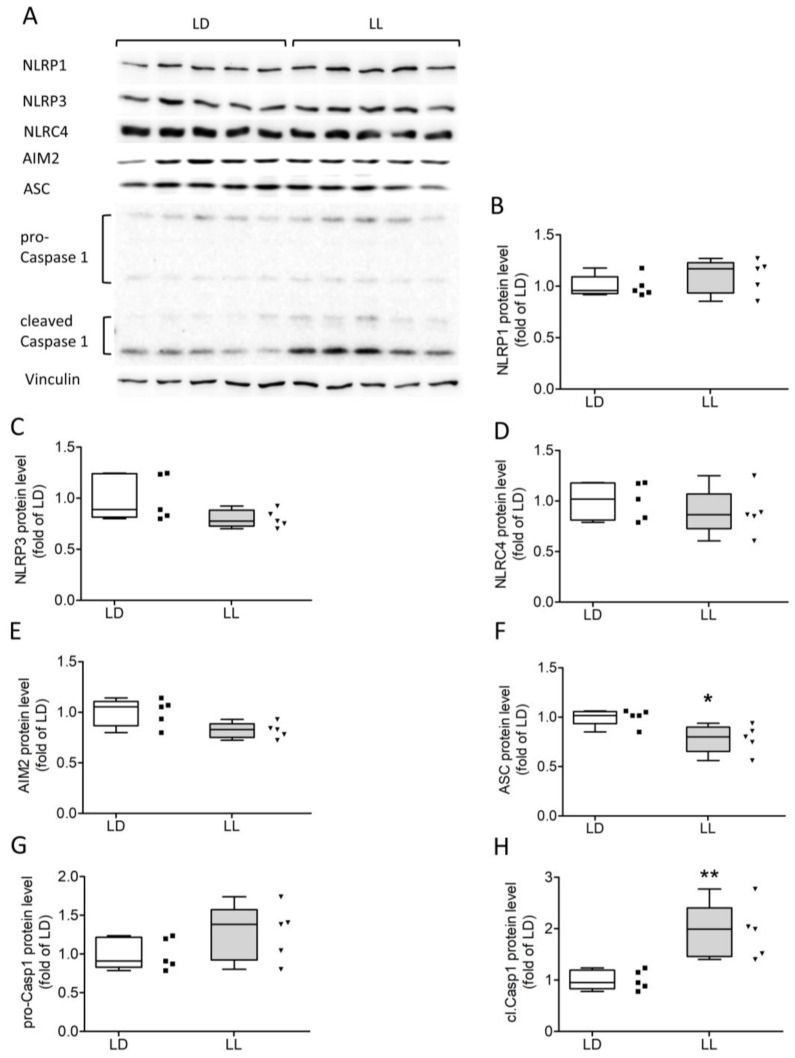
Protein concentration of active caspase 1 was significantly elevated after LL exposure. Western blot analysis of inflammasome components (**A**). Protein levels of ASC were reduced (**A**,**F**), whereas protein levels of cleaved caspase 1 (**A**,**H**) were significantly elevated in LL mice. No differences were found for NLRP1 (**A**,**B**), NLRP3 (**A**,**C**), NLRC4 (**A**,**D**), AIM2 (**A**,**E**), and pro-caspase 1 (**A**,**G**). Data were analyzed using Mann–Whitney U tests. * *p* < 0.05, ** *p* < 0.001.

Next, we investigated the effects of LL on inflammasome protein levels using Western blot (Figure 5). Levels of cleaved caspase 1 (Figure 5A,H) were significantly higher in the LL group (Mann–Whitney U = 0.00, n1 = n2 = 5, *p* = 0.0079 two-tailed). Relative levels of cleaved pro-caspase 1 were not different (Figure 5A,G). Relative protein levels of ASC were reduced in mice in LL (Figure 5A,F, Mann–Whitney U = 2.000, n1 = n2 = 5, *p* = 0.0317 two-tailed). Protein levels of the inflammasome senor proteins NLRP1 (Figure 5A,B), NLRP3 (Figure 5A,C), NLRC4 (Figure 5A,D), and AIM2 (Figure 5A,E) were not significantly different between both groups.

**Figure 6:**
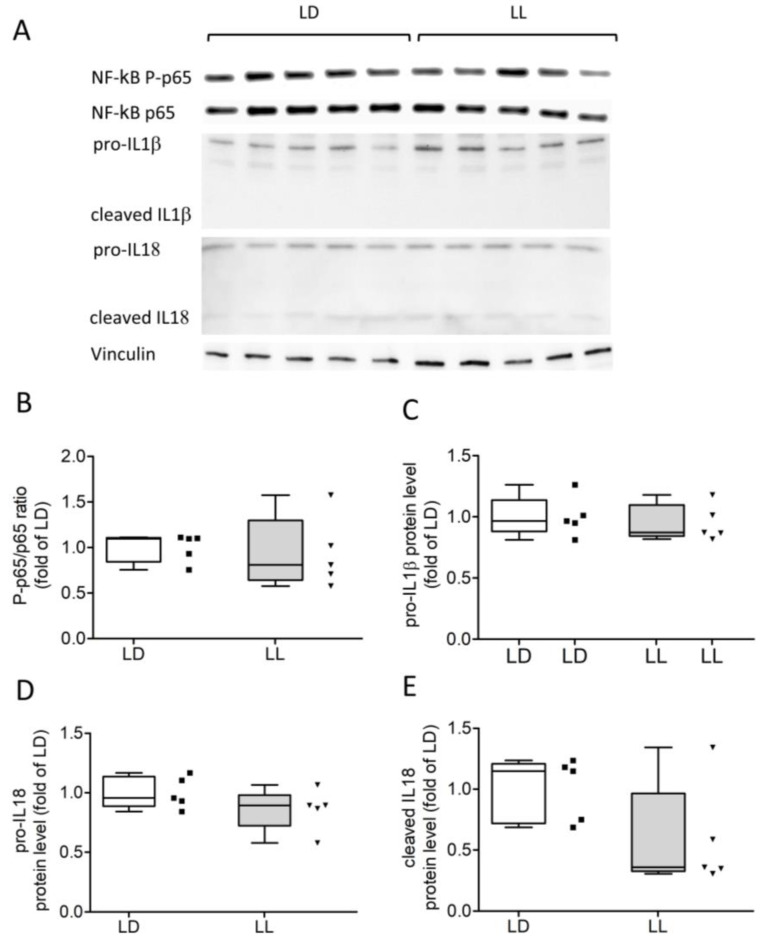
Protein levels of NF-κB, IL1β, and IL18 were unaffected by constant light. Western blot analysis of the P-p65/p65 ratio (**A**,**B**), interleukin IL1β (**A**,**C**), and IL18 (**A**,**D**,**E**) levels revealed no significant differences between LD and LL. Data were analyzed using Mann–Whitney U tests.

In the final step, we analyzed the phosphorylation of the transcription factor NF-ĸB and cytokines, including IL1β and IL18 (Figure 6A). There was no difference in the levels of the phosphorylated NF-ĸB subunit p65 (NF-κB P-65, Figure 6A,B), the cytokine precursors pro-IL1β (Figure 6A,C), pro-IL-8 (Figure 6A,D), or cleaved IL18 (Figure 6A,E) between LD and LL. Immunoreactive bands for cleaved IL1β were at the limit of detection (Figure 6A) and were, therefore, not quantified.

## 4. Discussion

In the present study, we analyzed the effects of LL on behavioral rhythms, weight, leukocyte composition, glial cells, and inflammasome expression in the hippocampus of healthy male C57BL/6 mice. Changes in rhythmic activity indicate that acute circadian disruption by constant light affects the circadian system. Total weight gain and white blood cell composition were not affected, suggesting that LL does not induce general systemic stress or inflammatory response. However, IBA1 immunoreaction was increased, and levels of cleaved caspase 1 were significantly elevated in the LL hippocampus, indicating microglia reactivity and inflammasome activation. On the contrary, inflammasome priming appears to be insufficient, as NF-κB-induced transcriptional expression of inflammasome components and cytokines was absent.

In modern society, artificial light at night, e.g., by shift work or night-time exposure to blue light-enriched LED screens, potentially inducing disruption of circadian rhythms, is highly prevalent. There is increasing evidence that circadian disruption is a risk factor for the development of metabolic, psychiatric, and neurological diseases. Importantly, this interaction is bidirectional, as many diseases negatively affect circadian rhythms [54].

As expected from the literature [55,56,57], LL exposure resulted in an increase in the period length and a reduction in total activity levels and strength of circadian rhythms. In accordance with a previous study [57], two weeks of LL did not result in increased weight gain or changes in peripheral blood leucocyte composition, which is consistent with the literature [57]. Earlier studies reported increased body weight gain in Swiss Webster mice, starting as early as one week after the onset of LL conditions [58,59,60]. The discrepancy between our observations and earlier studies might be multifactorial due to different mouse strains, sex, age, lighting conditions (hours of light cycle or light intensity: full light vs. dim light conditions), and others. For example, it was shown that body mass was increased under dim light at night exposure starting from adolescent (5–11 weeks) but not juvenile (3–9 weeks) developmental epochs. Importantly, weight and daytime food intake were increased in male but not female adolescent mice [61]. In a study by Kooijman et al., 2015, final body weight was not changed, but gonadal white adipose tissue weight and adipocyte size were significantly elevated after 5 weeks of LL [62]. Finally, pineal gland melatonin content and plasma melatonin concentration, and/or expression of melatonin receptors in different laboratory strains of mice [63,64] may further account for certain variability when studying circadian disruption in mice. However, our findings are in accordance with a recent study analyzing C57BL/6 mice under constant light for a time course of up to 24 weeks. Significant changes in body weight and leukocyte composition were observed after 8 but not 2 weeks of LL, indicating a stronger effect of chronic circadian disruption on metabolism and immune status [57].

It has been confirmed that the susceptibility of an organism to an immune stimulus (e.g., LPS challenge) is tightly controlled by the circadian system and that LL severely perturbs the inflammatory response [18,20,57,65]. Importantly, circadian misalignment (LL, jet lag, etc.) and stress, in general, have been shown to facilitate a pro-inflammatory state even under unchallenged conditions [47,66,67,68]. Moreover, the chronic stress-induced inflammasome-driven inflammatory response in the mouse hippocampus was followed by a depressive-like behavior [47]. In this study, we tested the hypothesis that LL for two weeks affects the brain inflammasome in the absence of an additional external stressor or immune stimulus. We noticed a slight but significant increase in IBA1 immunoreaction. However, GFAP expression and ARG1/NOS2 ratio were unchanged, merely suggesting a mild glial reactivity. Most importantly, we found significantly elevated levels of cleaved caspase 1 protein, the effector caspase activated through inflammasome assembly [69], under LL conditions. Though, transcription and protein levels of IL1β and IL18 were not affected under LL. A two-step activation mechanism has been proposed for the NLRP3 inflammasome: 1. Priming: DAMPs/PAMPs (e.g., LPS, TNF) interact with the appropriate membrane receptor, leading to NF-κB activation, nuclear translocation, and increased transcription of NLRP3 and pro-interleukins; 2. Inflammasome activation: On receiving a second signal (ATP influx, mitochondrial reactive oxygen species (mtROS), and others), cytosolic inflammasome sensors multimerize, and recruit ASC and pro-caspase 1, followed by caspase 1 activation and interleukin processing [70,71,72,73]. Additionally, posttranscriptional mediated priming, such as deubiquitination and phosphorylation, are essential for the formation of the NLRP3 inflammasome [74,75]. Transcriptional priming is required for subsequent IL1β processing and secretion since expression levels are very low or undetectable in healthy cells or tissue. Since levels of active caspase 1 (inflammasome assembly and activation step) but not phosphorylated NF-κB p65 (priming step) were elevated, we propose that acute circadian disruption promotes inflammasome assembly but not priming. However, our results are in accordance with previous studies, demonstrating NLRP3 activation in the absence of priming signals [76]. In a recent study by Vijayaraj et al., it was shown that pro-IL1β is rapidly turned over by K133A ubiquitylation and proteasomal degradation. Furthermore, ubiquitinated pro-IL1β becomes inaccessible by caspase 1 cleavage and exhibits a faster turnover compared to the inflammasome components NLRP3, ASC, and caspase 1 [77,78]. Thus, short half-life and ubiquitin-mediated degradation tightly control pro-IL1β levels independently of inflammasome activation. Among the PRRs, we found comparable levels of NLRP1, NLRP3, NLRC4, and AIM2 in the mouse hippocampus, but their expression was mostly unaffected by LL. Decreased mRNA levels of AIM2 and caspase 1, as well as reduced protein levels of ASC in LL, may indicate a negative feedback loop that attenuates caspase 1 activation and potentially damages inflammation. The potential DAMPs for inflammasome activation in response to LL, TNF, and ROS [70] are promising candidates as they are upregulated in circadian disruption [79,80].

Limitations of the present study are the small sample size and single endpoint point analysis after two weeks under LL, without considering circadian rhythms in inflammasome and cytokine expression. Additionally, the sole use of male mice in our setup did not allow for possible sex differences. Inflammasome components and cytokines, including NLRP1, NLRP3, caspase 1, and IL1β, are diurnally expressed in different tissues [42,81,82], and sex differences have been detected in various disease models [83,84,85]. Further studies of chronic circadian disruption inducing a systemic immune response [57] and larger cohorts including both sexes should show whether this produces a similar effect on the hippocampal inflammasome as other chronic stress [47].

## 5. Conclusions

In conclusion, acute circadian disruption induced by constant light seems to sense the innate immune system and, thus, promotes inflammasome assembly and cleavage of the inflammasome effector caspase 1. The absence of transcriptional activation of IL precursors by NF-κB, usually followed by increased cytokine processing, indicates inefficient priming. However, an active “ready-to-start” inflammasome platform may boost pro-inflammatory cytokine production if coupled with an additional harmful stimulus (e.g., infection, trauma, etc.) and thus may make the brain more vulnerable to inflammation, aging, and neurodegeneration.

## Figures and Tables

**Table 1:** List of primers.

Primer	Sequence (5′ to 3′)	
AIM2	Sense: Antisense:	GCAAAACAAAGTGCGAGGAA TTCAAGGAGCAGCATCAGGA
ARG1	Sense: Antisense:	CTCCAAGCCAAAGTCCTTAGAG AGGAGCTGTCATTAGGGACATC
ASC	Sense: Antisense:	CTTGTCAGGGGATGAACTCAAAA GCCATACGACTCCAGATAGTAGC
Caspase 1	Sense: Antisense:	CCGTGGAGAGAAACAAGGAGT CCCCTGACAGGATGTCTCCA
GFAP *	Sense: Antisense:	CGGAGACGCATCACCTCTG AGGGAGTGGAGGAGTCATTCG
GAPDH	Sense: Antisense:	AGGTCGGTGTGAACGGATTTG TGTAGACCATGTAGTTGAGGTCA
HSP90	Sense: Antisense:	TACTACTACTCGGCTTTCCCGT TCGAATCTTGTCCAGGGCATC
IBA1	Sense: Antisense:	ATCAACAAGCAATTCCTCGATGA CAGCATTCGCCTCAAGGACATA
IL18	Sense: Antisense:	TGCCAAAAGGAAGATGATGC ACACAAACCCTCCCCACCTA
IL1b	Sense: Antisense:	GACGGACCCCAAAAGATGAA TCCACAGCCACAATGAGTGA
NLRC4	Sense: Antisense:	ATCGTCATCACCGTGTGGAG GCCAGACTCGCCTTCAATCA
NLRP1b	Sense: Antisense:	AGCCCTCAAAGATGCCCCTT TTGTGTTCTCAGCCCGCACT
NLRP3	Sense: Antisense:	TGACCCAAACCCACCAGTGT TGTGCAGACCTCCCCAATGT
NOS2	Sense: Antisense:	ACATCGACCCGTCCACAGTAT CAGAGGGGTAGGCTTGTCTC

* PrimerBank database ID 30692526a1.

**Table 2:** List of primary antibodies.

Antibody	Host	Company, Order Number	WB	Target Size (kDa)
AIM2 ASC	Rabbit Rabbit	Bioss, Woburn, MA, USA, bs-5986R Adipogen, Fuellinsdorf, Switzerland, AG-25B-0006-C100	1:1000 1:1000	40 20
Caspase 1 GAPDH GFAP IBA1 IL18 IL1β	Mouse Mouse Goat Rabbit Rabbit Rabbit	Adipogen, Fuellinsdorf, Switzerland, AG-20B-0042-C100 Santa Cruz, Dallas, TX, USA, sc-32233 Abcam, Cambridge, UK, ab53554 Fujifilm Wako, Neuss, Deutschland, 019-19741 Santa Cruz, Dallas, TX, USA, sc-7954 Novus, CO, USA, NB600-633	1:1000 1:10,000 1:10,000 1:1000 1:1000 1:1000	20, 45 35 55 17 18, 24 17, 31
NLRC4 NLRP1 NLRP3 NF-κB p65 P-NF-κB p65 Vinculin	Rabbit Rabbit Rabbit Rabbit Rabbit Mouse	Merck, Darmstadt, Germany, 06-1125 Novus, CO, USA, NB100-56148 Bioss, Woburn, MA, USA, bs-10021R Cell signaling, Danvers, MA, USA, 8242S Cell signaling, Danvers, MA, USA, 3039S Santa Cruz, Dallas, TX, USA, sc-73614	1:1000 1:1000 1:1000 1:1000 1:1000 1:1000	116 136 118 65 65 116

## Data Availability

The data presented in this study are available on request to the corresponding author.

## Abbreviations

AD	Alzheimer’s disease
AIM2	Absent in melanoma 2
ALAN	Artificial light at night
ALS	Amyotrophic laterals sclerosis
ARG1	Arginase 1
ASC	Apoptosis-associated speck-like protein containing a CARD
DAMP	Damage-associated molecular pattern
FFT	Fast Fourier transform analysis
GAPDH	Glyceraldehyde-3-phosphate dehydrogenase
GFAP	Glial fibrillary acidic protein
HSP90	Heat shock protein 90
IBA1	Ionized calcium-binding adapter molecule 1
IL	Interleukin
NOS2	Nitric oxide synthase 2
LL	12:12 light/light, constant light
LPS	Lipopolysaccharides
mtROS	Mitochondrial reactive oxygen species
NF-κB	Nuclear factor kappa B
NLR	NOD-like receptor
PAMP	Pathogen-associated molecular pattern
PD	Parkinson’s disease
PRR	Pattern recognition receptor
ROS	Reactive oxygen species
Rev-erba	Nuclear receptor subfamily 1 group D member 1 (NR1D1)
SCN	Suprachiasmatic nucleus
TLR	Toll-like receptor
